# Hyperbaric Oxygen Therapy Reduces the Traumatic Brain Injury–Mediated Neuroinflammation Through Enrichment of Prevotella Copri in the Gut of Male Rats

**DOI:** 10.1007/s12028-024-01997-1

**Published:** 2024-05-15

**Authors:** Tee-Tau Eric Nyam, Hsiao-Yue Wee, Min-Hsi Chiu, Kuan-Chi Tu, Che-Chuan Wang, Yao-Tsung Yeh, Ching-Lung Kuo

**Affiliations:** 1 Department of Neurosurgery, Chi Mei Medical Center, 901 Chung Hwa Road, Yung Kang Dist., Tainan, 71004 Taiwan; 2 Department of Neurosurgery, Chi Mei Medical Center, Liouying, Tainan, Taiwan; 3 Aging and Disease Prevention Research Center, Fooyin University, Kaohsiung, Taiwan; 4 Department of Medical Laboratory Science and Biotechnology, Fooyin University, Kaohsiung, Taiwan; 5 Department of Medical Research, Chi Mei Medical Center, Tainan, Taiwan; 6 School of Medicine, Colledge of Medicine, National Sun Yat-Sen University, Kaohsiung, Taiwan; 7 Center of General Education, Chia Nan University of Pharmacy and Science, Tainan, Taiwan

**Keywords:** Traumatic brain injury, Neuro-inflammation, Tumor necrosis factor alpha, Gut microbiota, Brain–-gut axis

## Abstract

**Background:**

Gastrointestinal dysfunction frequently occurs following traumatic brain injury (TBI) and significantly increases posttraumatic complications. TBI can lead to alterations in gut microbiota. The neuroprotective effects of hyperbaric oxygen (HBO) have not been well recognized after TBI. The study’'s aim was to investigate the impact of HBO on TBI-induced dysbiosis in the gut and the pathological changes in the brain following TBI.

**Methods:**

Anesthetized male Sprague–Dawley rats were randomly assigned to three groups: sham surgery plus normobaric air (21% oxygen at 1 atmospheres absolute), TBI (2.0 atm) plus normobaric air, and TBI (2.0 atm) plus HBO (100% oxygen at 2.0 atmospheres absolute) for 60 min immediately after TBI, 24 h later, and 48 h later. The brain injury volume, tumor necrosis factor-α expression in microglia and astrocytes, and neuronal apoptosis in the brain were subsequently determined. The V3–V4 regions of 16S ribosomal rRNA in the fecal samples were sequenced, and alterations in the gut microbiome were statistically analyzed. All parameters were evaluated on the 3rd day after TBI.

**Results:**

Our results demonstrated that HBO improved TBI-induced neuroinflammation, brain injury volume, and neuronal apoptosis. HBO appeared to increase the abundance of aerobic bacteria while inhibiting anaerobic bacteria. Intriguingly, HBO reversed the TBI-mediated decrease in *Prevotella copri* and *Deinococcus* spp., both of which were negatively correlated with neuroinflammation and brain injury volume. TBI increased the abundance of these gut bacteria in relation to NOD-lik0065 receptor signaling and the proteasome pathway, which also exhibited a positive correlation trend with neuro inflammation and apoptosis. The abundance of *Prevotella copri* was negatively correlated with NOD-like receptor signaling and the Proteasome pathway.

**Conclusions:**

Our study demonstrated how the neuroprotective effects of HBO after acute TBI might act through reshaping the TBI-induced gut dysbiosis and reversing the TBI-mediated decrease of *Prevotella copri*.

## Introduction

Traumatic brain injury (TBI) remains a public health problem, often resulting in long-term neurological complications [1, 2]. Neuroinflammation, characterized by the activation of astrocytes and microglia leading to the release of tumor necrosis factor -α (TNF-α), has been observed following TBI and may play a significant role in the complications of increased brain injury volume and neuronal apoptosis [3]. These complications typically manifest by the 3rd day after the injury and can serve as a time point to assess the injury in the acute phase [4].

Several basic and clinical studies provide supporting evidence that hyperbaric oxygen (HBO) has neuroprotective effects in TBI. The mechanisms include increasing dissolved oxygen in the plasma, enhancing recovery of damaged mitochondria, improving aerobic metabolism, and subsequently decreasing cerebrospinal fluid lactate levels [5, 6]. HBO is also known to increase vasoconstriction and alleviate cerebral edema [7]. It further reduces brain inflammation by suppressing astromicroglial activation and the expression of the proinflammatory cytokine TNF-α [8], while increasing the expression of anti-apoptotic proteins Bcl-2 and Bcl-xL [9].

Our previous studies have demonstrated that treatment with HBO (2.0 atmospheres absolute [ATA] in 100% O_2_, 60 min), once per day for 1 or 3 days, significantly attenuates local and systemic production of the proinflammatory cytokine TNF-α and neuronal apoptosis [10–12]. However, despite its inhibition of TNF-α production and prevention of intestinal mucosa apoptosis in an intestinal ischemia–reperfusion injury animal model [13], the effects of HBO on the gut after TBI have not been investigated. Therefore, further investigation is warranted to understand HBO-associated effects on the gut after TBI.

Recently, evidence has indicated that the bidirectional communication axis between the gut and the brain mediates inflammation after TBI [14]. After-TBI, gastrointestinal dysfunction can frequently occur [15, 16]. TBI can lead to significant damage to the structure of the intestinal mucosa, impairment of barrier function, and endotoxin production, peaking at 72 h [17]. These factors can significantly impact posttraumatic morbidity and mortality [18]. Furthermore, TBI may induce alterations in gut microbial populations in mice [19], and disruption of the gut microbiota has been linked to cardiovascular disease, inflammatory bowel disease, obesity, Parkinson’'s disease, and autism [20]. The possible mechanisms inking brain function and the intestinal microbiome form a feedback loop in which each component affects the other [19]. This implies that changes in gut microbial compositions can, in turn, influence the neurological system via feedback through the brain–-gut axis, potentially affecting outcomes following TBI. Therefore, reshaping the composition of the gut microbiome could serve as a potential therapeutic target.

To date, metagenomic sequencing, which uses 16S DNA sequencing of fecal samples extracted and polymerase chain reaction (PCR)-amplified techniques, has enabled the characterization of the human microbiome beyond the capacity of traditional culture techniques. It has identified the role of microbiota in normal physiological, immunological, and metabolic functions [21, 22]. Many microbial alterations were observed at 3 days after-TBI and correlated with a peak in magnetic resonance imaging lesion volume and loss of behavioral function. Moreover, a larger brain lesion was associated with greater decreases in the abundance of Firmicutes, an exacerbated increase in Proteobacteria, and a significant reduction in alpha diversity [22]. However, relatively few studies have been conducted focusing on the impact of TBI on the gut microbiome, and the clinical implications are still not well -established [22]. Furthermore, the impact of post-TBI treatment with HBO on the gut microbiome and the implications for functional outcomes remain unseen.

In the present study, we hypothesized that post-TBI treatment with HBO alters the gastrointestinal microbiome correlating with structural and functional characteristics of the brain, with a focus on neuroinflammation. To test this hypothesis, using 2.0 atm brain percussion, we investigated regional microglia and astrocyte activation, TNF-α expression, neuronal apoptosis, and brain injury volume in the brain. Additionally, we examined gut microbial populations and metabolic pathway changes on the 3rd day after TBI. Ultimately, our study demonstrated that the neuroprotective effects of HBO after acute TBI might act through reshaping TBI-induced gut dysbiosis.

## Materials and Methods

### Experimental Design

#### Traumatic Brain Injury

**Fig. 1:**
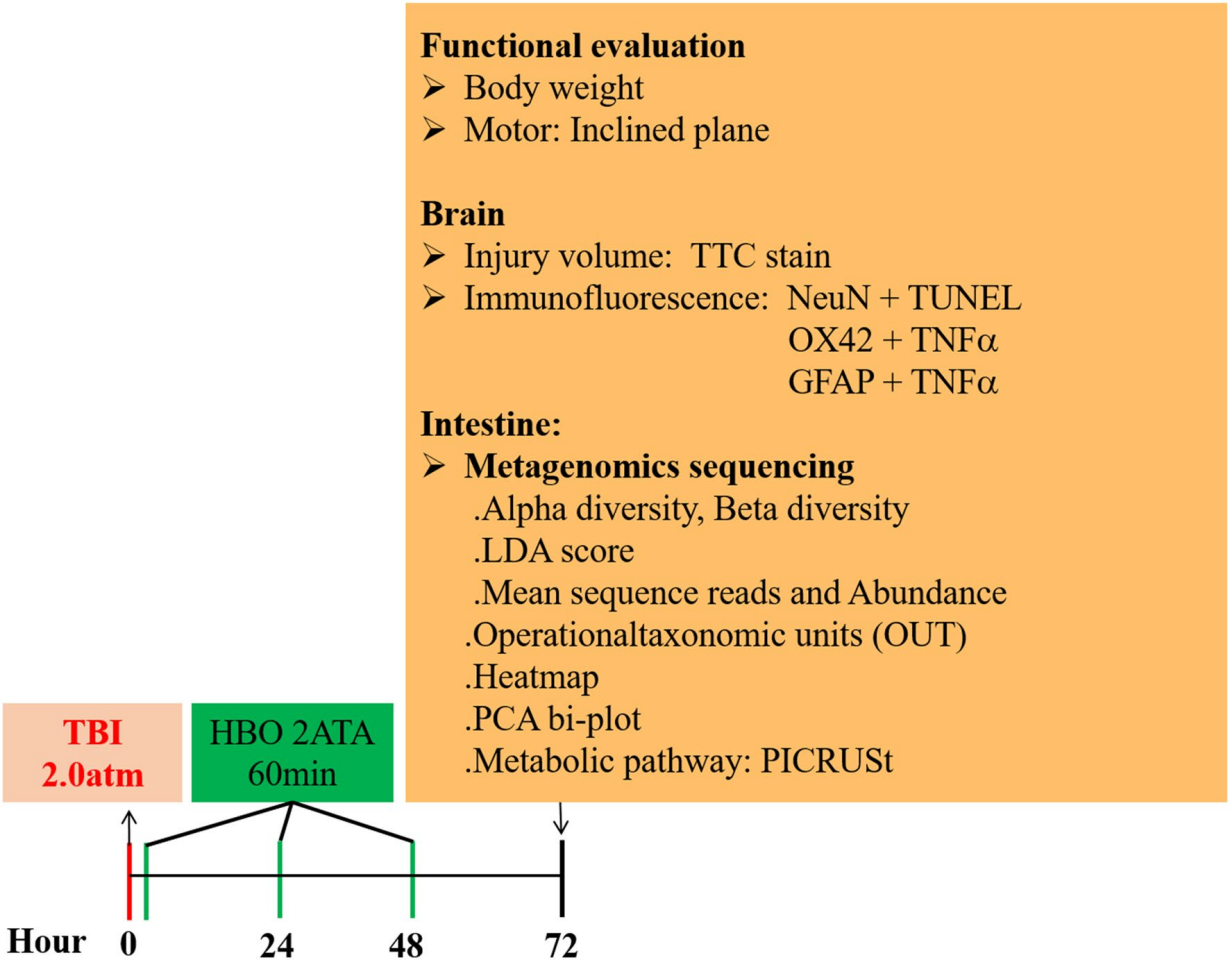
The overall experimental protocol in the current study. ATA, atmospheres absolute, GFAP, glial fibrillary acidic protein, HBO, hyperbaric oxygen, LDA, liner discriminant analysis, NeuN, neuronal nuclear antigen, OX42, monoclonal antibody OX42 for microglia, PCoA, principail coordinated analysis, PICRUSt, Phylogenetic Investigation of Communities by Reconstruction of Unobserved States, TBI, traumatic brain injury, TNFα, tumor necrosis factor α, TTC, triphenyltetrazolium chloride, TUNEL, terminal deoxynucleotidyl transferase–mediated biotin–deoxyuridine triphosphate nick-end labeling

The entire experimental procedure is depicted in Fig. 1. A total of 36 male Sprague–Dawley adult rats were employed in this study. All tests were conducted with the researchers being masked to the study groups, and group assignments were only revealed at the end of the analysis. Adequate food and water were consistently provided to the animals throughout the duration of the study. On completion of the experiment, the rats were humanely euthanized using an overdose of urethane. All experimental procedures were approved by the Chi Mei Medical Centre Animal Care and Use Committee and adhered to National Institutes of Health guidelines (no. 110122310).

### Animals

The animals were anesthetized by intraperitoneal injection of a mixture of ketamine (IM, 44 mg/Kg, i.m.; Nam Kwong Pharmaceutical, Taiwan), atropine (0.0633 mg/kg, i.m.; Suntong Chemical Industry Co., Ltd., Taiwan), and xylazine (6.77 mg/kg, i.m.; Bayer, Germany). Craniectomy with a radius of 2 mm was performed on a stereotaxic frame at 4 mm from the bregma and 3 mm from the sagittal suture of the right parietal cortex. After removal of the skull and implantation of the injury cannula, a fluid percussion device (VCU Biomedical Engineering, Richmond, VA) was attached to the animal via a luer lock connector, and the brain was injured with an impact force of 2.0 atm and 25 ms. This produced moderate brain trauma, as originally described by Mclntosh et al. [23]. Transient hypertensive responses, apnea, and seizures were observed immediately after fluid percussion injury, and they were used as criteria for successful TBI insult in rats. After the impact on the experimental rats, their tongues were immediately pulled out of the mouth to ensure the patency of the airway. There were no additional fatalities in the entire experimental setup because of this procedure.

### Surgery and Physiological Parameter Monitoring of the Brain

Under ketamine anesthesia, the right femoral artery of rats was cannulated with a polyethylene tube (PE50) for blood pressure monitoring. Colon temperature was measured using an electronic thermometer (Model 43TE; YSI Corporation, Yellow Springs, OH) and a temperature probe (400 series; YSI Corporation). Core temperature was maintained within the range of 36–37 °C using lamp insulation. All recordings were conducted on a four-channel Gould polygraph.

### Treatment Intervention

Animals were randomly numbered and assigned to different study groups using a random number table. The animals were then allocated to three groups (*n* = 12 in each group): sham surgery plus normobaric air (NBA) (21% oxygen at 1 ATA), TBI (2.0 atm) plus NBA (21% oxygen at 1 ATA), and TBI (2.0 atm) plus HBO (100% oxygen at 2.0 ATA). Animals designated for HBO treatment (100% oxygen at 2.0 ATA) underwent a 60-min treatment immediately after TBI, as well as at 24 h and 48 h after post-TBI. Animals in the NBA group were exposed to room air, whereas those in the HBO group were placed in the HBO chamber. The chamber was flushed with 100% oxygen at a rate of 5 l/min to prevent CO accumulation. Decompression was carried conducted at a rate of 0.2 kg/cm^2^/min. The temperature of the pressure chamber was maintained between 22 and 25 °C.

Detailed information on the numbers of animals and results analysis is provided in Table 1.

**Table 1:** Detailed information on the numbers of animals and results analysis

Group	Parameter	
	Body weight inclined plane TTC stain	Metagenomics sequencing IF stain
Sham plus normobaric air (21% oxygen at 1 ATA)	72 h after craniectomy (*n* = 6)	72 h after craniectomy (*n* = 6)
TBI (2.0 atm) plus normobaric air	72 h after TBI (*n* = 6)	72 h after TBI (*n* = 6)
TBI (2.0 atm) plus hyperbaric oxygen (100% oxygen at 2.0 ATA)	72 h after TBI (*n* = 6)	72 h after TBI (*n* = 6)

ATA, atmospheres absolute, IF, immunofluorescence, TBI, traumatic brain injury, TTC, triphenyltetrazolium chloride

### Cerebral Injury Volume Assay

The staining procedure for triphenyltetrazolium chloride (TTC) was conducted following the protocol described elsewhere. All animals were euthanized on the 3rd day after fluid percussion injury. Under deep anesthesia with ketamine, animals were intracardially perfused with saline. The brain tissue was then removed, immersed in cold saline for 5 min, and sliced into 1-mm sections. These brain sections were incubated in a 2% TTC solution dissolved in phosphate-buffered saline at 37 °C for 30 min and sequently transferred to a 10% formaldehyde solution for fixation. The injury volume, indicated by negative staining for TTC (reflecting dehydrogenase-deficient tissue), was assessed for each section using computer planimetry (computer-based image tool software) and summarized. The injury volume was calculated as 1 mm (thickness of the slice) multiplied by the sum of injury areas (in square millimetersmm2) in all brain sections [24].

### Neuronal Apoptotic Assay in Neuronal CELLS in the Peri-Injury Cortex Using Iimmunofluorescence Stain

Apoptotic neuronal cells were identified by terminal deoxynucleotidyl transfer-ase-mediated biotin–deoxyuridine triphosphate nick-end labeling (TUNEL) [25] staining on day 3 after TBI. These procedures followed those described previously [26]. The quantity of immunofluorescence assay–-positive cells was determined corresponding to the peri-injury cortex (magnification × 400). The results were expressed as the mean number of positive cells in all five sections from each rat, using computerized planimetry (Image-Pro Plus, Media Cybernetics, Inc., Rockville, MD). The following anti-bodies were used in this study: monoclonal mouse anti-NeuN antibody (NeuN, Millpore, MAB377) at 1:100 dilution, followed by Alexa-Fluor, 568 anti-mouse (IgG) antibody (Invitrogen, A11031) at 1:400 dilution.

### Microglial, Astrocyte Activation in the Peri-Injury Cortex Using Iimmunofluorescence Assay

On day 3 after TBI, activated microglia were assessed by detecting OX42-positive cells using an immunofluorescence assay [27]. The number of OX42 cells in the sample was measured in each slice and summed using a computerized planar measurement (Image Tools software based on PC-Q4Q5). Monoclonal mouse anti-OX42 antibody (ab78457; Abcam, Boston, MA), anti-glial fibrillary acidic protein (GFAP) antibody (MA5-15,086; Thermo USA) and polyclonal goat anti-TNF-α antibody (sc-1351; Santa Cruz Biotechnology Inc, Santa Cruz, CA) was used in this study and used Alexa-Fluor, 568 antimouse (IgG) antibody (A11031; Life Technologies Co, Grand Island, NY), and DyLight 488 antigoat (IgG) antibody (ab96931; Abcam), respectively.

### Fecal DNA Extraction

The genomic DNA was extracted from the fecal samples by using the QIAmp Fast DNA Stool Mini Kit (Qiagen, Germany) with the modified instructions. Briefly, the stool sample was centrifuged at 13,200 rpm for 10 min to remove the storage buffer and lysis with the InhibitEX buffer. Add the proteinase K and ethanol to obtain the processed su-pernatant. Finally, the supernatant was washed with the QIAamp spin column and eluted with the elution buffer. The concentration was assessed by NanoDrop 2000 and then performed × 10X dilution with elution buffer.

### Next Generation Sequencing Analysis

The gut microbiome library was constructed with the standard V3–V4 region of the 16S ribosomal RNA gene. PCR amplification was performed with KAPA HiFi hotstart readymix (Roche) and purified using AMPure XP magnetic beads (Beckman Coulter, Brea, CA). The amplified PCR products were assessed for quality using a Fragment Analyzer (Advanced Analytical, Wood County, WV) and quantified using a Qubit 3.0 Fluorometer. Subsequently, the library was sequenced on a MiSeq platform (Illumina, San Diego, CA) with paired-end reads (2 × 300 nt), generating at least 100,000 reads for each sample.

### Bioinformatics Analysis and Statistics

The raw paired‐end reads were be trimmed, and that pass the quality filters assigned to operational taxonomic units which ≥ 97% similarity with the Greengene Data-base (v13.8). Operational taxonomic units (relative abundance), alpha diversity, beta diversity (PCoA), and heatmap were performed with the MicrobiomeAnalyst (https://www.microbiomeanalyst.ca/MicrobiomeAnalyst/upload/OtuUploadView.xhtml), CLC genomics workbench (Qiagen, Germany), and GraphPad Prism 8 (GraphPad Software, La Jolla, CA). The abundance bacteria analysis (Linear discriminant analysis Effect Size [LEfSe]) and functional analysis (Phylogenetic Investigation of Communities by Reconstruction of Unobserved States) were performed by Galaxy/Hutlab website (http://huttenhower.sph.harvard.edu/galaxy/).

### Statistical Analysis

All of the data were analyzed using SigmaPlot, version 10.0 for Windows (Systat Software, San Jose, CA) in this study. Results are expressed as the means ± standard deviations of the mean for the experiments. The *t*-test, Spearman’s correlation, one-way analysis of variance, and post hoc analysis were used to compare the group differences. A *p* value less than 0.05 was considered statistically significant. The SPSS program was used for the above calculations.

## Results

### Treatment of TBI with HBO During the Acute Phase of Injury Reduces TBI-Induced Brain Injury Volume, Neuroinflammation, and Neuronal Apoptosis

**Fig. 2:**
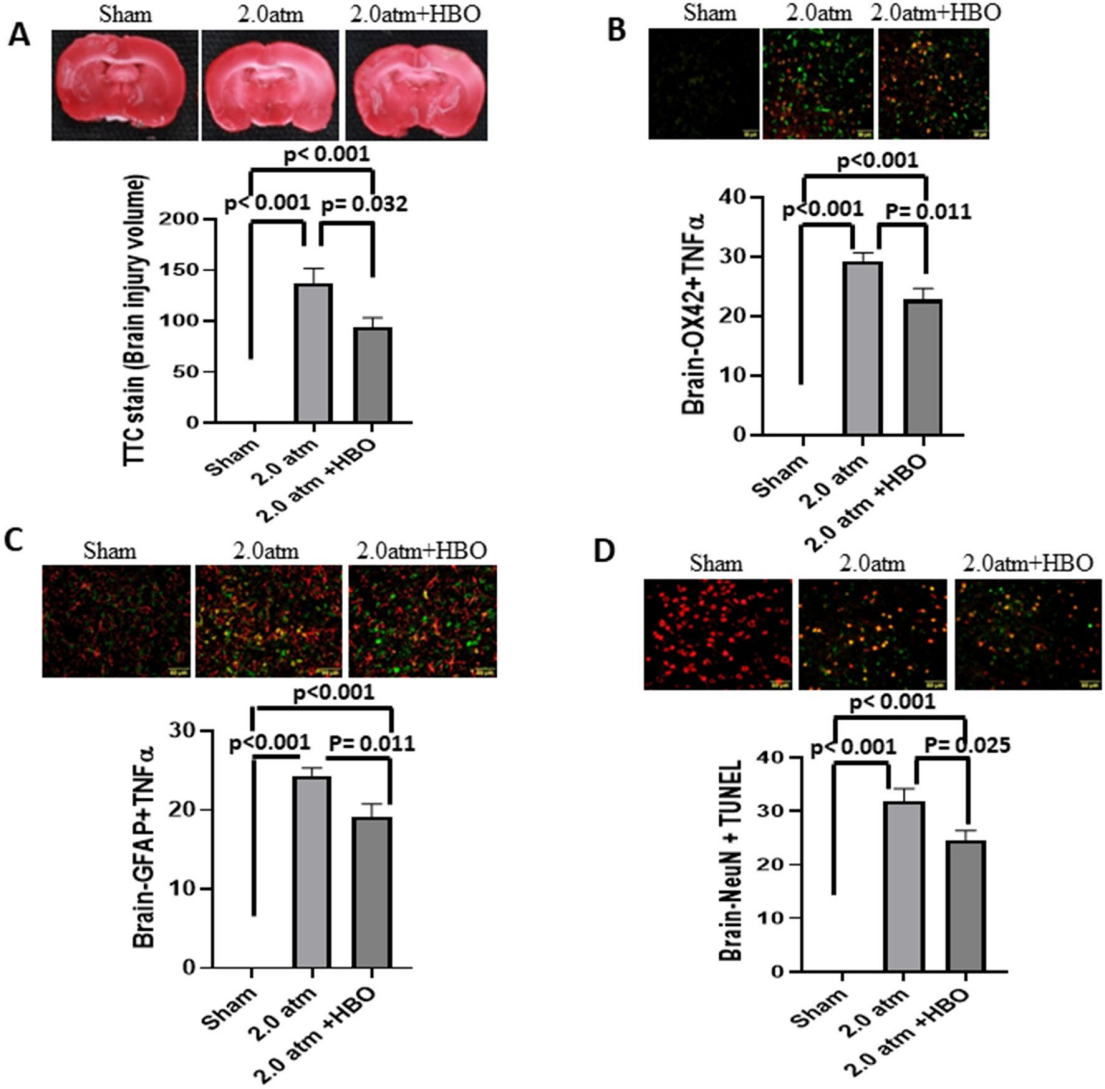
Treatment of TBI with hyperbaric oxygen therapy in the acute phase of injury reduces neuronal apoptosis, brain injury volume and TNF-α expression in the brain. **A** The brain injury volume was showed by TTC stain (**A**); the neuroinflammation marker were showed in (**B**) Brain-OX42 + TNFα and (**C**) Brain-GFAP + TNFα; the neuronal apoptosis marker was showed in (**D**) Brain-NeuN + TUNEL (*n* = 6 in each group). GFAP, glial fibrillary acidic protein, HBO, hyperbaric oxygen, NeuN, neuronal nuclear antigen, OX42, monoclonal antibody OX42 for microglia, TBI, traumatic brain injury, TNFα, tumor necrosis factor α, TTC, triphenyltetrazolium chloride, TUNEL, terminal deoxynucleotidyl transferase–mediated biotin–deoxyuridine triphosphate nick-end labeling

**Fig. 3:**
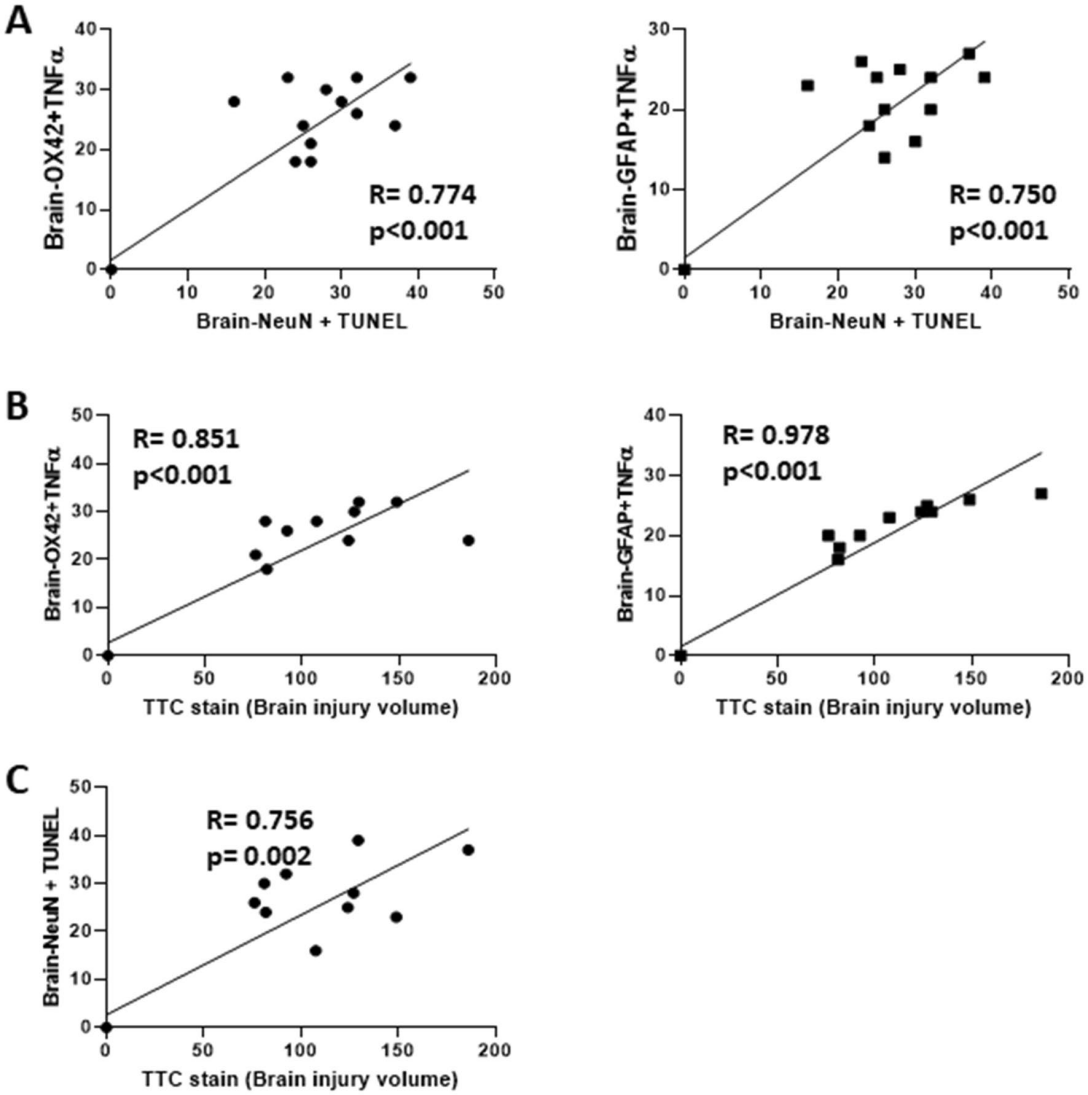
The correlation among neuroinflammation, brain injury volume and neuronal apoptosis was analyzed by using Spearman correlation in (**A**) neuroinflammation (Brain-OX42 + TNFα and Brain-GFAP + TNFα) and neuronal apoptosis (Brain-NeuN + TUNEL); (**B**) neuroinflammation (Brain-OX42 + TNFα and Brain-GFAP + TNFα) and brain injury volume (TTC stain); (**C**) neuronal apoptosis (Brain-NeuN + TUNEL) and brain injury volume (TTC stain) (*n* = 6 in each group). GFAP, glial fibrillary acidic protein, HBO, hyperbaric oxygen, NeuN, neuronal nuclear antigen, OX42, monoclonal antibody OX42 for microglia, TNFα, tumor necrosis factor α, TTC, triphenyltetrazolium chloride, TUNEL, terminal deoxynucleotidyl transferase–mediated biotin–deoxyuridine triphosphate nick-end labeling

At 72 h after TBI, TTC-stained sections revealed a significant increase in brain injury volume in the NBO-treated TBI group (136 ± 13 cm^3^) compared with sham TBI controls (0 ± 0 cm^3^, ***p < 0.001, *n* = 6 in each group). However, TBI-induced brain injury volume was significantly reduced by HBO treatment (94 ± 8 cm^3^, *p* = 0.045, *n* = 6 in each group, see Fig. 2A). The neuroinflammation markers Brain OX42 + TNF-α (Fig. 2B) and Brain-GFAP + TNF-α (Fig. 2C) also exhibited a significant increase in the brain injury volume of the NBO-treated TBI group, which was effectively reversed by HBO treatment following TBI. Furthermore, Fig. 1D illustrates that TBI-induced neuronal apoptosis marker Brain Neu-N + TUNL was significantly mitigated by HBO treatment following TBI (*n* = 6 in each group). Importantly, there exists a statistically significant positive correlation between neuroinflammation, brain injury volume, and neuronal apoptosis (see Fig. 3).

### Treatment of TBI with HBO did not Change Total Species and Compositions of Gut Microbiomes

**Fig. 4:**
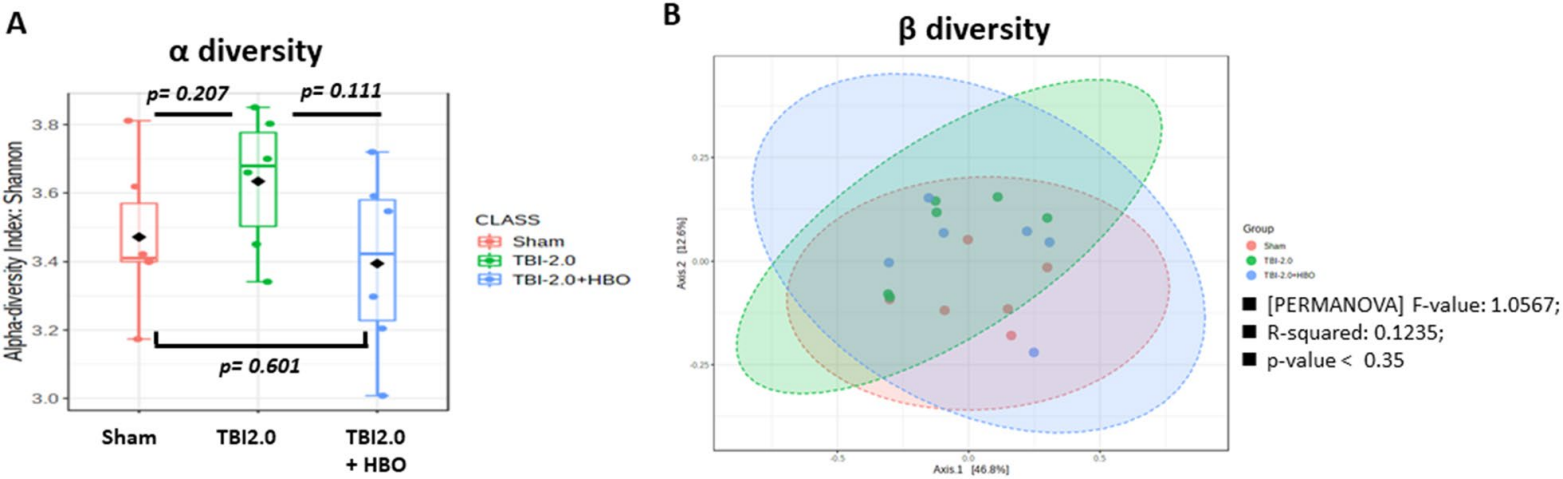
The total species diversity ([a] alpha diversity; **A**) and [b] bacteria composition ([beta diversity]; **B**) were analyzed by. Microbiome Analyst. TBI and HBO did not change total species and compositions of gut microbiomes (*P* < 0.35), (*n* = 6 in each group). HBO, hyperbaric oxygen, TBI, traumatic brain injury

On the 3rd day after moderate TBI treatment (TBI 2.0 atm), we used online tools, specifically alpha and beta diversity analysis via Microbiome Analyst, to assess the diversity of the microbiota. We found that the total species diversity (alpha diversity) showed no significant changes: (Sham VS TBI 2.0, *p* = 0.207; TBI 2.0 VS TBI 2.0 + HBO, *p* = 0.111; sham VS TBI 2.0 + HBO, *p* = 0.691) (see Fig. 4A). Additionally, the overall bacterial composition (beta diversity) did not exhibit significant differences among the groups (see Fig. 4B).

**Fig. 5:**
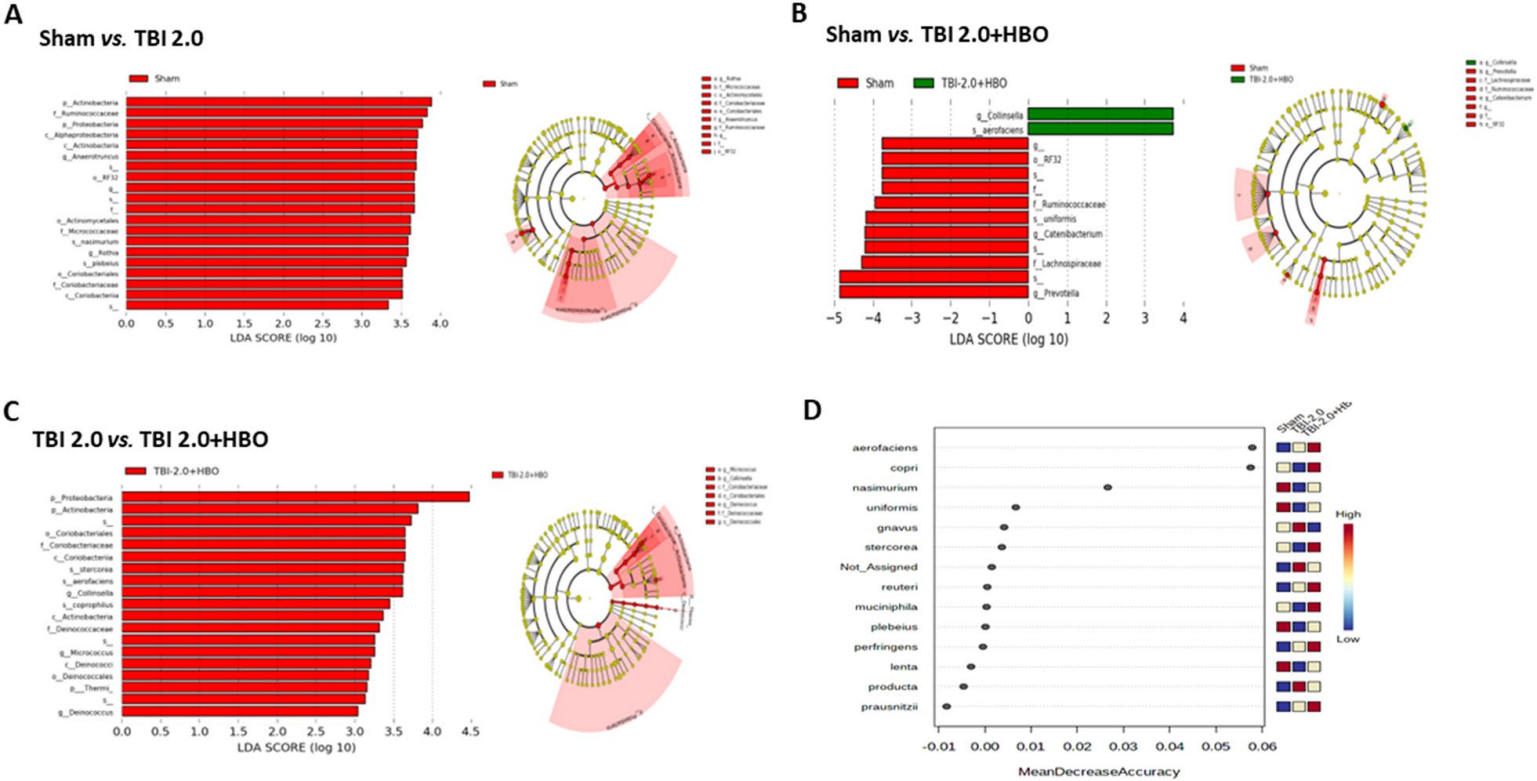
Using the LEfSe assay (Galaxy HutLab) to analysis the core abundant bacteria in (**A**) Sham vs. TBI 2.0 (*n* = 6 in each group); **B** Sham vs. TBI 2.0 + HBO (*n* = 6 in each group); **C** TBI 2.0 vs. TBI 2.0 + HBO, (*n* = 6 in each group). **D** We rank the importance of abundance bacteria in probiotic supplements by Random Forester analysis forester analysis. The importance of the abundant gut bacteria participated in the species. The top two gut microbe were *Collinsella aerofaciens* and *Prevotell copri*. HBO, hyperbaric oxygen, TBI, traumatic brain injury

We further used online tools, including LEfSe and Galaxy HutLab, to analyze the compositions of the core gut microbiome. We observed 14 abundant bacteria in the sham vs. TBI 2.0 group (Fig. 5A), 8 abundant bacteria in the sham vs. TBI 2.0 + HBO group (Fig. 5B), and 16 abundant bacteria in the TBI 2.0 vs. TBI 2.0 + HBO group, *n* = 6 in each group (Fig. 5C). Additionally, we employed an online Random Forest tool (Random Forester Analysis, Microbiome Analyst) to rank the importance of variables in a regression or classification problem. Based on the Random Forester analysis, our results indicated the *Collinsella aerofaciens* and *Prevotella copri* might be the most important gut microbes in HBO treatment for brain trauma (Fig. 5D).

**Fig. 6:**
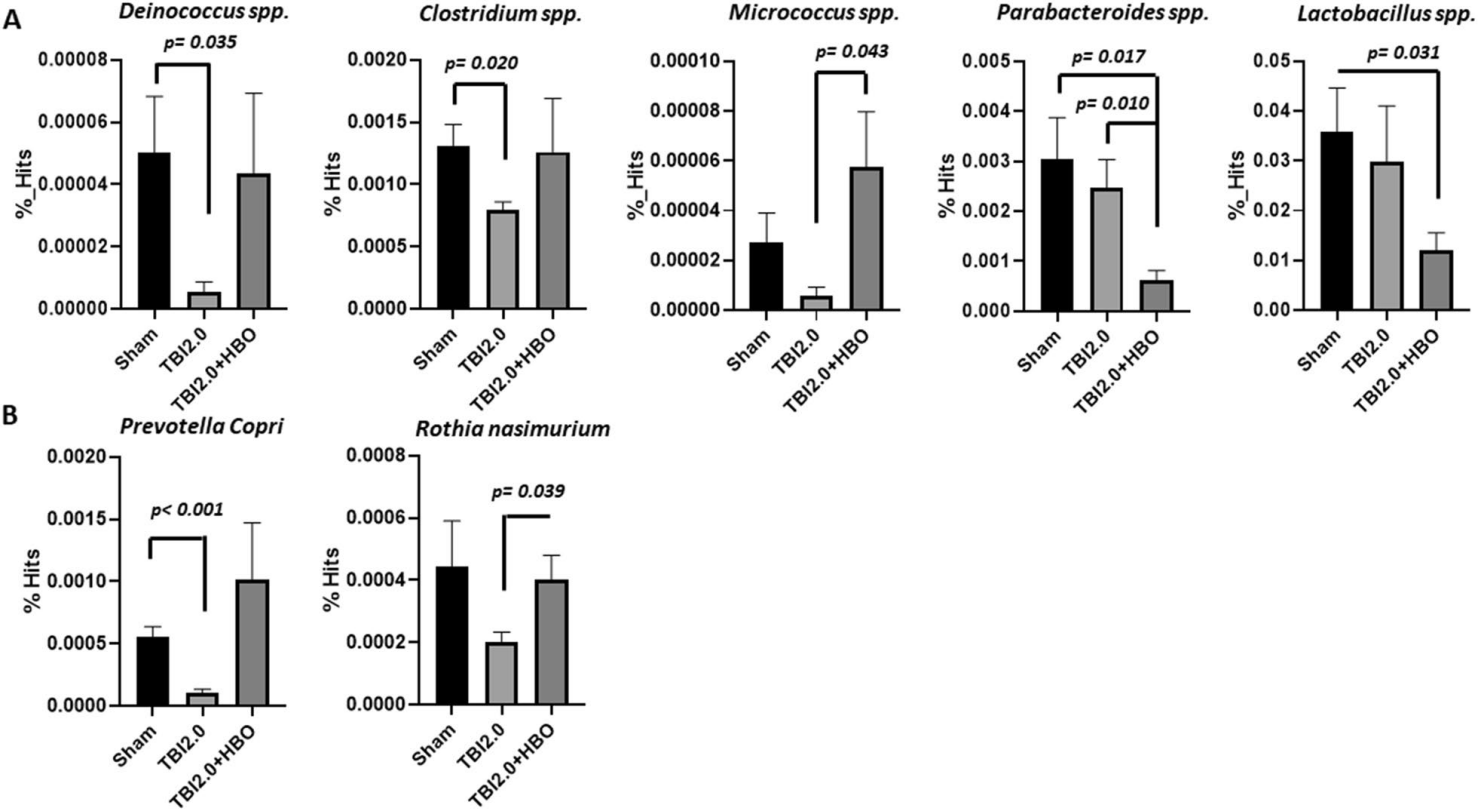
There were five5 abundance bacteria generaus and two2 abundance bacteria species among three groups. The abundance of *Prevotella copri* and *Deinococcus* spp. significantly decreased after brain injury and was reversible with HBO treatment. Data are presented as mMeans ± SEMs. Statistical comparison difference were determined by Student’s *t*-test. (*n* = 6 in each group). HBO, hyperbaric oxygen, SEM, standard error the of mean, TBI, traumatic brain injury

Based on the LEfSe analysis (Fig. 5A–C), we identified five abundant bacterialgenera (*Deinococcus* spp., *Clostridium* spp., *Micrococcus* spp., *Parabacteroides* spp., and *Lactobacillus* spp.) and two abundant bacterial species (*Prevotella copri* and *Rothia nasimurium*) among the three groups. Further analysis revealed that *Deinococcus* spp., *Clostridium* spp., and *Prevotella copri* were significantly decreased following brain trauma treatment (TBI 2.0) (*n* = 6 in each group). In contrast, HBO treatment (TBI 2.0 + HBO) significantly increased the abundance of *Micrococcus* spp. and *Rothia nasimurium* (Fig. 6).

### *Prevotella copri *and *Deinococcus* spp. Exhibited a Significant Negative Correlation with TTC stain, Brain-GFAP + TNFα, and Brain-OX42 + TNFα

**Fig. 7:**
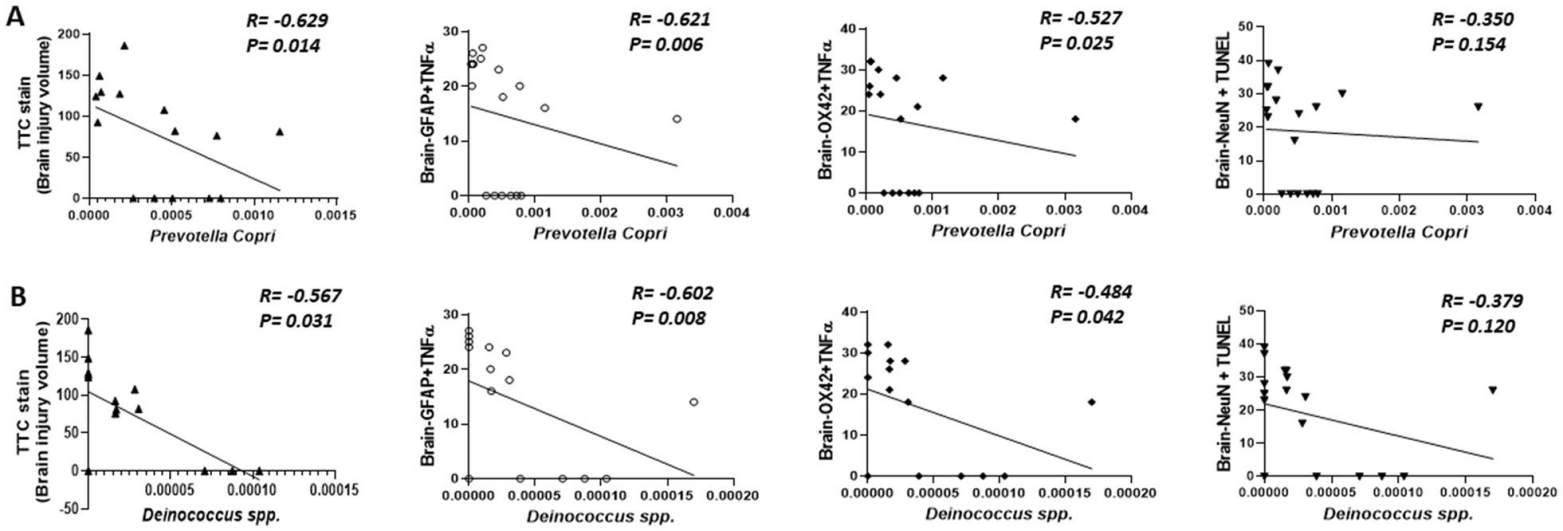
*Prevotella copri* and *Deinococcus* spp. had a significant negative correlation with brain injury volume, neuroinflammation and neuronal apoptosis. The abundance of *Prevotella copri* (**A**) and *Deinococcus* spp. (**B**) displayed correlations with brain injury volume, neuroinflammation and neuronal apoptosis. The correlation was analyzed by Spearman’s correlation analysis. (*n* = 6 in each group)

We analyzed the correlation abundant bacteria and brain injury volume, neuroinflammation, and neuronal apoptosis following TBI and treatment with HBO using Spearman’s correlation analysis. Our results revealed that *Prevotella copri* and *Deinococcus* spp. exhibited a significant negative correlation with TTC stain, Brain-GFAP + TNFα, and Brain-OX42 + TNFα (Fig. 7A, B) (*n* = 6 in each group). Additionally, both *Prevotella copri* and *Deinococcus* spp. showed a positive correlation trend with neuronal apoptosis.

### TBI Significantly Decreased Glutamatergic Synapse, Galactose Metabolism, and Sphingolipid Metabolism, and Significantly Increased NOD-like Like Receptor Signaling Pathway and Proteasome Pathway

**Fig. 8:**
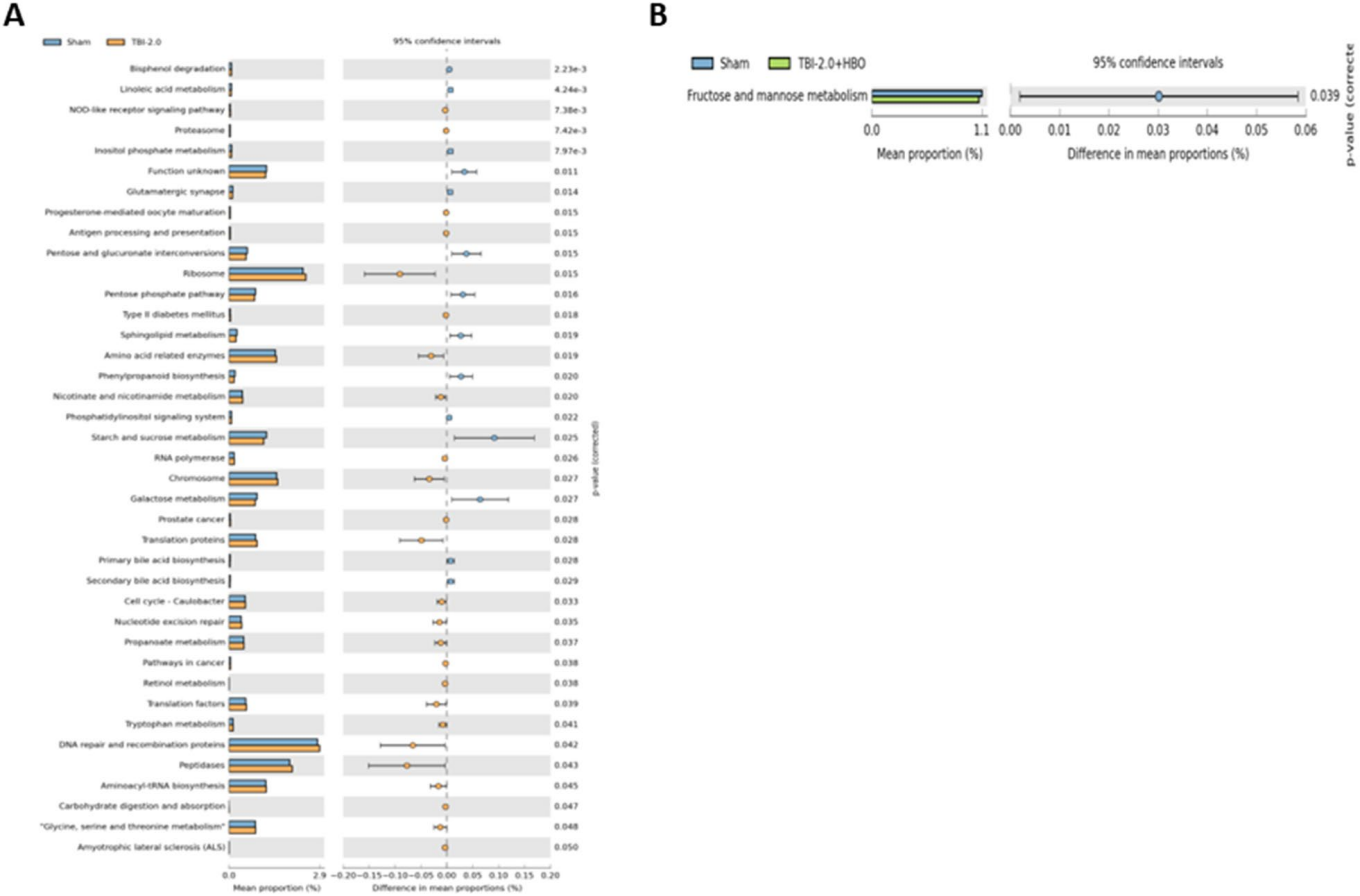
The functional analysis of gut microbiomes after TBI and HBO treatment. Comparison of the predicted function of gut microbiota taxa (**A**) between the Sham and TBI2.0 groups and (**B**) between the Sham and TBI2.0 + HBO groups. The functional analysis of gut microbiomes was analyzed by PICRUSt assay (Galaxy/HutLab). (*n* = 6 in each group). HBO, hyperbaric oxygen, PICRUSt, Phylogenetic Investigation of Communities by Reconstruction of Unobserved States, TBI, traumatic brain injury

We used online tools (Phylogenetic Investigation of Communities by Reconstruction of Unobserved States, Galaxy/HutLab) to analyze the effects of abundant bacteria on metabolic pathways among three groups. Our analysis revealed 39 functional pathways in the sham and TBI2.0 group (Fig. 8A), while only one functional pathway was identified in the sham and TBI2.0 + HBO group (Fig. 8B) (*n* = 6 in each group).

**Fig. 9:**
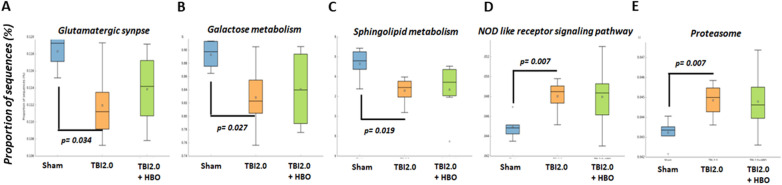
These trauma-induced functional pathways changes might be reversed by HBO therapy analyzed by PICRUSt assay (Galaxy/HutLab). **A** Glutamatergic synapse; **B** Galactose metabolism; **C** Sphingolipid metabolism; **D** NOD- like receptor signaling pathway; and **E** Proteasome pathway. (*n* = 6 in each group). HBO, hyperbaric oxygen, NOD, xxx, PICRUSt, Phylogenetic Investigation of Communities by Reconstruction of Unobserved States, TBI, traumatic brain injury

Furthermore, our results disclosed that TBI significantly decreased neuronal function in the glutamatergic synapse (*p* = 0.034), Galactose metabolism (*p* = 0.027), and sphingolipid metabolism pathways (*p* = 0.019). Additionally, TBI significantly modulated immune response pathways such as the NOD-like receptor signaling pathway (*p* = 0.007) and protein degradation pathways like the Proteasome (*p* = 0.007) (Fig. 9A–E) (*n* = 6 in each group). These trauma-induced effects might be reversed by HBO therapy.

### Glutamatergic Synapse, Galactose Metabolism, and Sphingolipid Metabolism had a Significant Negative Correlation with Neuronal Cell Apoptosis.

**Fig. 10:**
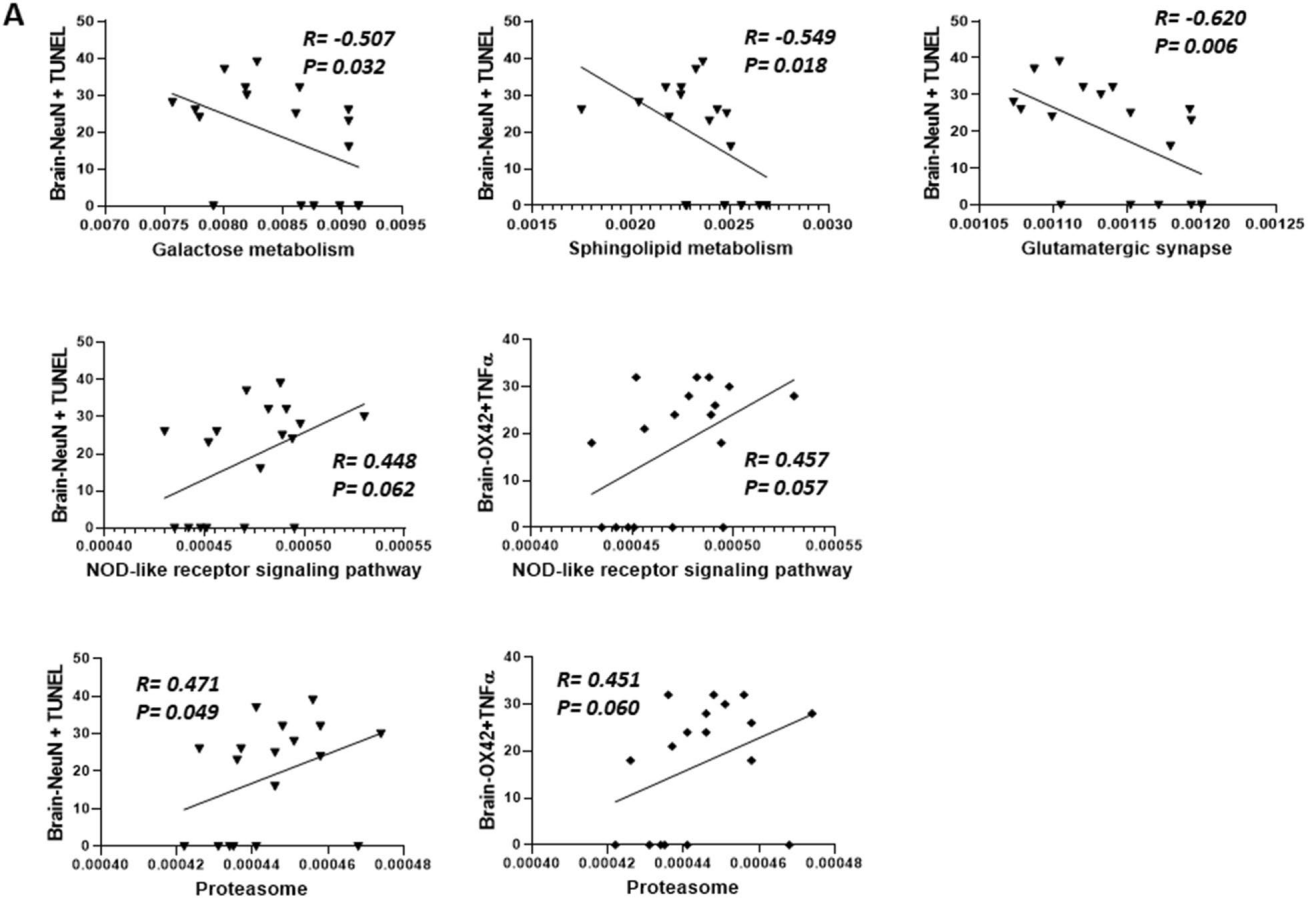
The Spearman’s correlation analysis to analyze the correlation of between the neuroinflammation, neuronal apoptosis and the metabolic pathway of bacteria after TBI treatment. (*n* = 6 in each group). TBI, traumatic brain injury

We conducted Spearman’s correlation analysis to explore the correlation between neuroinflammation, neuronal apoptosis, and bacterial metabolic pathways following TBI treatment (see Fig. 10). Our findings revealed that Glutamatergic synapse (R = − 0.607, *p* = 0.011), Galactose metabolism (R = − 0.487, *p* = 0.049), and Sphingolipid metabolism (R =  − 0.549, *p* = 0.024) exhibited a significant negative correlation with neuronal apoptosis (Brain-NeuN + TUNEL). Both the NOD-like receptor signaling pathway (R = 0.475, *p* = 0.056) and proteasome (R = 0.475, *p* = 0.056) displayed a positive correlation trend with neuronal apoptosis. Additionally, we observed a positive correlation trend between both the NOD-like receptor signaling pathway (R = 0.475, *p* = 0.056) and the proteasome (R = 0.475, *p* = 0.056) and neuroinflammation (*n* = 6 in each group).

### *Prevotella copri* Exhibited a Significant Negative Correlation with NOD-like Signaling Pathway and Proteasome

**Fig. 11:**
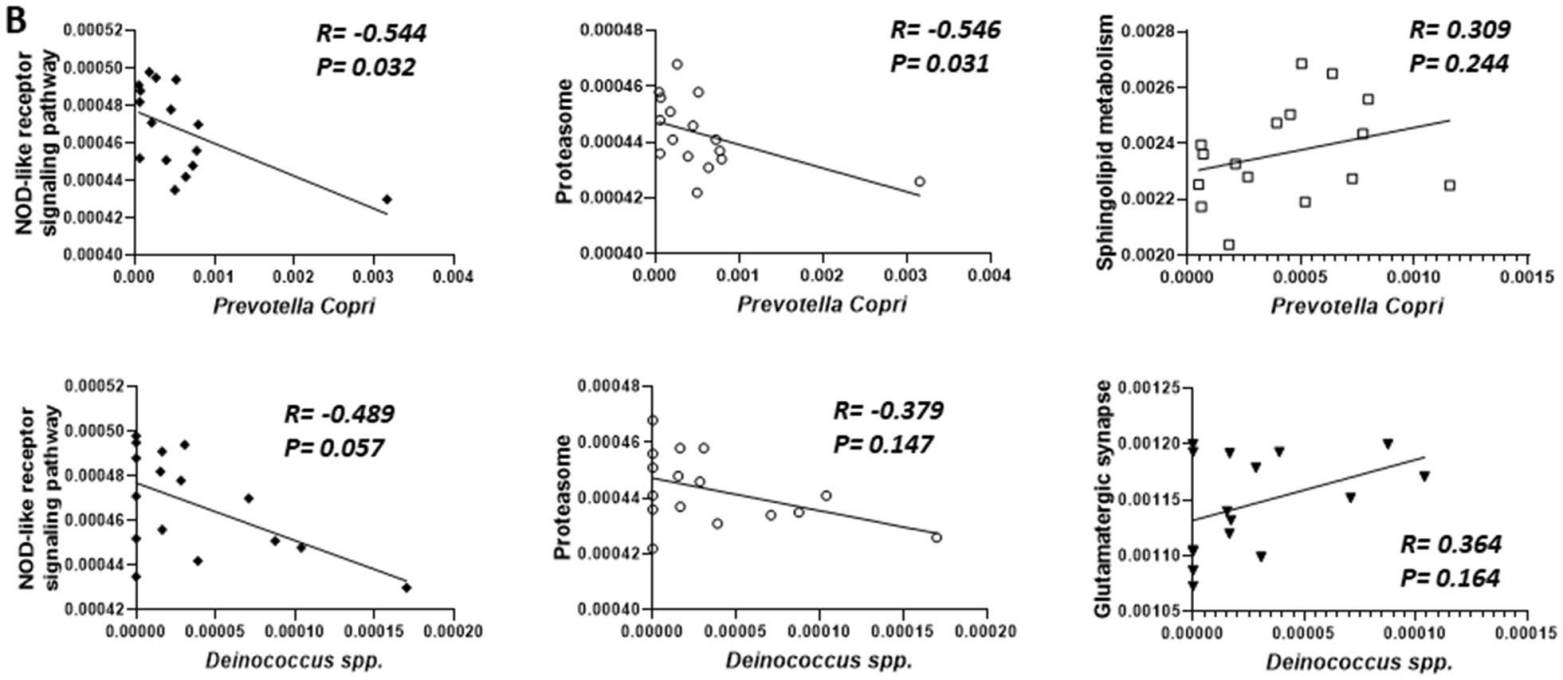
Analysis ofze the correlation between the abundant bacteria and the metabolic pathways of abundant bacteria using the Spearman’s correlation analysis. (*n* = 6 in each group)

Finally, we conducted Spearman’s correlation analysis to examine the correlation of between abundant bacteria and their metabolic pathways (Fig. 11). *Prevotella copri* exhibited a significant negative correlation to with the NOD-like signaling pathway (R =  − 0.544, *p* = 0.032) and the proteasome (R =  − 0.546, *p* = 0.031)., However, in contrast, it showed a modest positive correlation with sphingolipid metabolism, specifically in neuronal function (R = 0.309, *p* = 0.244) (*n* = 6 in each group).

## Discussion

### Novelty of Current Study

According to our literature review, our study is the first to demonstrate that HBO therapy could influence the gut microbiota composition after TBI, and TBI-induced neuroinflammation may correlate with the abundance of *Prevotella copri* and the NOD-like signal pathway. We believe that these findings lay the foundation for future microbiota research under TBI and HBO treatment conditions.

### TBI and HBO Effects on Brain

Consistent with previous studies, our results demonstrate that HBO has beneficial effects on TBI-induced neuroinflammation, apoptosis, and brain injury volume [8, 10]. Additionally, we present new findings indicating a significant positive correlation between neuroinflammation and brain damage volume or neuronal apoptosis (see Fig. 3). These findings suggest that neuroinflammation plays a crucial role in the injury process following TBI and highlight the inhibition of inflammatory responses as a potential target for brain injury treatment.

### TBI and HBO Effects on Core Gut Bacterial Composition

When considering the gut bacterial composition on the 3rd day after TBI, our findings on regarding alpha diversity revealed a nonsignificant increase after TBI (see Fig. 4). Interestingly, this result contrasts with significant gut microbiota depletion observed in other animal models after TBI [22, 28, 29]. We hypothesize that this discrepancy may be attributed to differences in the nature of the impact injuries. For instance, our study used fluid impacts with a force of 2.0 atm, whereas other studies employed direct impacts using a pneumatic impactor (5.0 m/s; 250 μs dwell time and 2 mm depth), or impacts induced by placing a 50 g load at a height of 10 cm and loosely applying the impacts along the guide rails [28, 29]. These varying impact mechanisms may result in differential effects on the intestinal flora. Therefore, further investigations are warranted to elucidate whether HBO therapy exerts distinct effects on gut microbiota composition in response to different levels of brain injury.

Evidence has shown that TBI could alter some gut bacterial taxa. Treangen et al. [30] demonstrated that there was an early decrease in genus-level or species-level abundance of bacterial gut microbiome 24 h after the experimental TBI model. Rogers et al. [31] have shown early depletion of commensal bacteria and enrichment of potential pathogens at 72 h after TBI in pediatric severe TBI. In the current study, we found that five genus-level and two species-level bacteria of core gut bacteria decreased at 72 hours after TBI. These TBI-induced bacterial depletion effects could be further altered is determined by whether the bacteria themselves are aerobic or anaerobic through HBO therapy (Fig. 5). These results are consistent with HBO therapy being able to alter the composition of the gut microbiota [32].

Being as anaerobic bacteria, the abundance of *Parabacteroides* spp. and *Lactobacillus* spp. were significantly decreased after HBO therapy. In contrast, as aerobic bacteria, the abundance of *Micrococcus* spp. and *Rothia nasimurium* increased. This result is consistent with Wu et al. [33] report that increased tissue oxygenation could directly influence microbes, such as reducing *Anaerostipes*. Because 90% of the intestinal tract is composed of anaerobic bacteria [34], HBO therapy should be helpful in the development of pathologies caused by the proliferation of anaerobic bacteria.

### TBI and HBO Effects on *Prevotella copri*

An increase or decrease in *Prevotella copri* levels compared to normal values will influence disease progression. Increased levels of *Prevotella copri* have been reported in patients with new-onset untreated rheumatoid arthritis [35] and irritable bowel syndrome [36], while decreased levels have been detected in patients with neurodegenerative diseases [37]. Consistent to with findings in neurodegenerative diseases, our current study revealed a significant decrease in *Prevotella copri* levels after TBI, which could be reversed after HBO therapy (Fig. 6). Additionally, its level was found to be significantly negatively correlated with neuroinflammation and brain injury volume (Fig. 7). These results suggest that using HBO therapy to repair gut dysbiosis caused by TBI may represent a novel mechanism for reducing inflammation in the brain.

### TBI and HBO Effects on Metabolic Pathway

The glutamatergic synaptic pathway is interconnected with numerous other neurotransmitter pathways and is pivotal in a wide array of normal physiological functions [38]. A delicate equilibrium between sphingolipid synthesis and degradation is typically essential for various biological processes [39, 40], and alterations in their metabolism could significantly impact brain homeostasis and function. Nonetheless, the detailed mechanism requires further clarification in future studies.

### The Reduction of TBI-Induced Neuroinflammation by *Prevotella copri* may be Related to the Inhibition of NOD-Like Receptor Signaling and the Proteasome Pathway

NOD-like receptor has been reported to Nod1 as novel regulators for the stress response, 5-HT biosynthesis, and signaling. These results further indicate that Nod1 may contribute to a previously unrecognized signaling pathway in the gut-brain axis [41]. NOD-like receptor C5 promotes positively regulate neuroinflammation, suppresses neuronal survival and dopaminergic degeneration in Parkinson’s disease (PD), and may serve as a marker of glial activation [42]. In this study, we identified a positive correlation trend between the NOD-like receptor signaling pathway and the proteasome with neuronal apoptosis and neuroinflammation. Finally, we observed a significant negative correlation between *Prevotella copri* levels and the NOD-like signaling pathway and proteasome activity. Therefore, we suggest that attenuating TBI-induced neuroinflammation may be related to the inhibition of NOD-like receptor signaling and proteasome pathways, and may also be part of the neuroprotective mechanism of HBO. However, this speculation needs to be further clarified in the future.

### Limitations of Current Study

There are some limitations to our study. Firstly, it is important to address the width of the therapeutic window for HBO treatment. In the current study, the clinical significance of initiating HBO treatment immediately after impact was found to be limited. In future studies, we plan to delay the first HBO exposure until 3, 6, 12, or 24 h after TBI to enhance clinical relevance and comprehensively assess the effect of HBO on TBI. Second, we employed three groups to assess the effects of HBO on TBI in rats, adhering to our established study design from previous research. there were only three groups of animals: sham + normal oxygen, TBI + normal oxygen and TBI + hyperbaric oxygen. It would be more beneficial to compare normobaric 100% oxygen to HBO at 2 ATA and normobaric room air at 21% oxygen. This comparison would help determine whether there is any additional benefit from HBO compared to the interventions clinicians can attempt without placing the patient in an HBO chamber. In addition, without a control group with a sham procedure and exposure to hyperbaric oxygen, one cannot determine whether the change of gut bacteria is related to hyperbaric oxygen or the response to both TBI and hyperbaric oxygen. Third, we only examined the effect of short-term HBO interventions after injury, whereas a 3-day assessment is evident for assessing parameters related to neuroinflammation in secondary injury due to TBI [8]. Fourth, we used only moderate fluid impact injuries (2.0 atm) to assess the effects of HBO on the brain and gut. Further studies are needed to determine whether HBO has a differential effect on the severity of brain injury. Fifth, although OX42 has been identified as a marker for microglia, it is crucial to acknowledge that, in addition to microglia, OX42 also stains for granulocytes. Likewise, although TUNEL staining has been established as a marker for apoptotic cells, it may produce positive results in necrotic and autolytic cells, as well. Therefore, a more specific marker is considered essential for future studies. Sixth, we did not assess histopathologic changes in the gut although it is now known that gut damage occurs after TBI [15]. Finally, importantly, *Prevotella Copri* abundance was negatively correlated with NOD-like receptor signaling and proteasome pathways. However, the causal relationship between *Prevotella copri* and NOD-like receptor signaling and proteasome pathways remains unknown. In addition, it is unclear whether supplementation with HBO, *Prevotella copri* ameliorates TBI-mediated neuroinflammation and apoptosis. Therefore, more studies are needed to explore the above relationships and potential therapeutic effects.

## Conclusions

Our study suggests that HBO therapy can influence gut microbiota composition after TBI. The neuroprotective effects of HBO after TBI may be mediated by remodeling TBI-induced dysbiosis of the gut flora and reversing TBI-mediated reduction of *Prevotella copri*.

## Data Availability

Authors declare that under request, all qualitative and quantitative data will be shared.
